# Electroextraction of Insoluble Proteins from the Organic Matrix of the Nacreous Layer of the Japanese Pearl Oyster, *Pinctada fucata*

**DOI:** 10.3390/mps2020037

**Published:** 2019-05-09

**Authors:** Daisuke Funabara, Natsumi Miyashita, Kiyohito Nagai, Kaoru Maeyama, Satoshi Kanoh

**Affiliations:** 1 Graduate School of Bioresources, Mie University, Kurimamachiya 1577, Tsu 514-8507, Japan; natsumimiyashita262@gmail.com (N.M.); kanoh@bio.mie-u.ac.jp (S.K.); 2 Pearl Research Laboratory, K. MIKIMOTO & CO., LTD., Osaki Hazako 923, Hamajima, Shima 517-0403, Japan; k-nagai@mikimoto.com; 3 Mikimoto Pharmaceutical CO., LTD., Kurose 1425, Ise 516-8581, Japan; maeyama.511@mikimoto-cosme.com

**Keywords:** electro-extracted proteins, electroextraction, heating method, nacreous layer, organic matrix, pearl oyster

## Abstract

The nacreous layer of shells and pearls is composed of aragonite crystals arranged in an organic matrix. The organic matrix contains chitin and several proteins that regulate the formation of the nacreous layer. Owing to their strong interactions in the organic matrix, the current method for extraction of insoluble proteins from the pre-powdered nacreous layer involves heating to high temperatures in the presence of a detergent (e.g., sodium dodecyl sulfate, SDS) and reductant (e.g., dithiothreitol, DTT), which is likely to induce protein degradation. Therefore, we have developed an electroextraction method to isolate proteins from the organic matrix of a nacreous organic sheet, that was obtained following the decalcification of shells in their original shape. Our electroextraction method employs milder conditions without heating or detergent. Sodium dodecyl sulfate-polyacrylamide gel electrophoresis (SDS-PAGE) patterns of the electro-extracted proteins (EEPs) under non-reduced and reduced conditions revealed that this method yielded a greater number of different proteins compared with the conventional extraction method and the isolated EEPs retained their disulfide bonds. Our method is able to easily extract insoluble proteins from the nacreous layer under mild conditions and will undoubtedly aid future analyses into the functions of the nacreous layer proteins.

## 1. Introduction

The Japanese pearl oyster, *Pinctada fucata*, is one of the most important molluscan species in the pearl culture industry. These oysters form pearls using the same process that produces the nacreous inner layer of their shells: Mantle tissue and a nucleus implanted into an oyster body cause the formation of a pearl sac, which subsequently closes. Over time, nacreous layers are repeatedly secreted on the surface of the nucleus that eventually results in the formation of a pearl [1]. Formation of the nacreous layer in *P. fucata* shells is thought to be regulated by several types of proteins that are secreted from the mantle tissue. To date, despite extensive studies over many years, the molecular mechanism of nacreous layer formation remains unclear [2]. A substantial part of previous investigations has focused on the functional characterization of purified protein components isolated from the nacreous layer.

The nacreous layer is composed of aragonite tablets arranged in an organic matrix comprised of chitin that forms compartments filled with silk-like proteins associated with acidic glycoproteins [3]. In the compartments, it is thought that the aragonite tablets mature from the primary colloidal amorphous calcium carbonate nanoparticles [4]. This process is considered to be regulated by an amorphous calcium carbonate-binding protein [5,6]. The sheet-like organic matrix is exposed by decalcifying the nacreous layer of shells with ethylenediamine-*N*,*N*,*N*′,*N*′-tetraacetic acid (EDTA) or various acids. Although some proteins are eluted during the decalcification step, many others are retained in the organic matrix. Unfortunately, harsh conditions are required to extract the retained proteins from the organic matrix, as they appear to strongly interact with each other. For example, extraction of the aragonite binding protein, Pif80, required heat treatment at 100 °C for 10 min in the presence of SDS and DTT, following shell decalcification with acetic acid [7]. The putative shell framework proteins, MSI31 and MSI60, were isolated through decalcification of the nacreous layer with methanoic acid, followed by cyanogen bromide treatment [8]. Finally, N16, an aragonite forming protein, was extracted from the nacreous matrix with an alkaline solution of NH_4_OH, following shell decalcification using 10% EDTA [9].

Here, we developed a novel method to electro-extract nacreous layer proteins under milder conditions, which enabled the extraction of proteins that are cross-linked by intermolecular disulfide bonds.

## 2. Materials and Methods

### 2.1. Decalcification of the Nacreous Layer

We obtained cultured *P. fucata* specimens (two years old) from Ago Bay, Mie Prefecture, Japan. The nacreous layers of shells were exposed by removing the prismatic layer with an angle grinder. The nacreous layers were decalcified in 0.5 M EDTA (Dojindo Laboratories, Kumamoto, Japan) (pH 8.0) for 14 days at 4 °C. The remaining organic matrix was named the nacreous organic sheet (NOS).

### 2.2. Heat Extraction of Proteins from the NOS

The NOS (30 mg) was mixed with 200 µL of 2× SDS sample buffer (20 mM Tris-HCl, pH 6.8, 2% SDS, 2% 2-mercaptoethanol, 40% glycerol, 4 mM EDTA, and 0.015% bromophenol blue) then boiled for 2 min, sonicated for 10 min, and boiled again for 2 min. After the addition of an equal volume of Milli-Q water, the heat-extracted proteins (HEPs) were used for subsequent analyses.

### 2.3. Electroextraction of Proteins from the NOS

**Figure 1:**
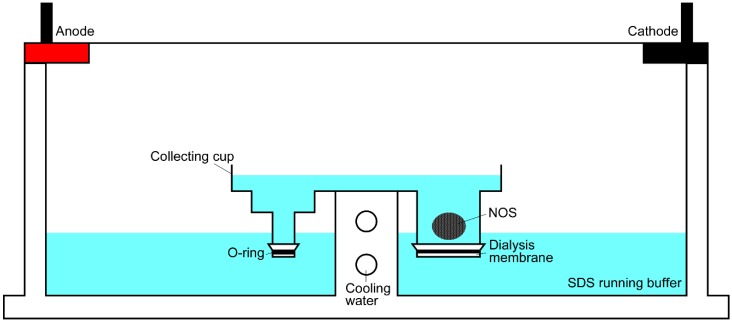
Diagram showing the electroextraction apparatus used to extract proteins from the nacreous organic sheet. NOS: nacreous organic sheet.

Proteins were extracted from the NOS using a NA-1710 protein recovery apparatus (Nihon Eido Co., Tokyo, Japan), which is usually used to electro-extract excised protein bands from SDS-PAGE gels. Dialysis membranes were attached to the bottom of the anode and cathode chambers of a sample cup using plastic O-rings to prevent any leakage of extracted proteins (Figure 1). The NOS (30 mg) was placed on the bottom of the cathode chamber and the sample cup and electrophoresis tank were filled with SDS running buffer (25 mM Tris, 192 mM glycine, and 0.1% SDS). Electroextraction was carried out for 3 h at 100 V. The apparatus is able to be cooled by running water throughout the electroextraction when necessary. However, the electroextraction chamber was not cooled in this study. Following the electroextraction process, the buffer in the anode cup was gently removed from the top of the chamber to approximately 1 cm from the bottom of the anode cup. This was performed using a pipette to prevent any buffer disturbances in the cup. The electro-extracted proteins (EEPs) were then carefully collected from the bottom of the anode cup to prevent puncturing of the dialysis membrane. The EEPs were dialyzed overnight against Milli-Q water, freeze-dried, and then resuspended in 200 µL of Milli-Q water.

### 2.4. SDS-PAGE Analysis

SDS-PAGE analysis was used to compare the protein composition of the EEPs with the HEPs prepared using the method described in Section 2.2. The EEP samples (40 µL) were mixed with an equal volume of 2× SDS-sample buffer and boiled for 2 min prior to analysis by SDS-PAGE, using 12% gels prepared with WIDE RANGE Gel Preparation Buffer (4X) for PAGE (Nakalai Tesque, Kyoto, Japan) and Coomassie blue staining. The band patterns for the HEP and EEP samples were then compared.

EEPs were analyzed by SDS-PAGE under non-reduced and reduced conditions to investigate if they contained disulfide bonds. A non-reduced sample was prepared as follows: EEPs (40 µL) were mixed with an equal volume of 2× SDS-sample buffer without 2-mercaptoethanol. The reduced samples were prepared by mixing an EEP sample (40 µL) with an equal volume of 2× SDS-sample buffer containing 2% 2-mercaptoethanol. Both samples were then boiled for 2 min prior to SDS-PAGE analysis and Coomassie blue staining. The band patterns of the reduced and non-reduced EEP samples were subsequently compared.

## 3. Results

### 3.1. Decalcification of the Pearl Oyster Shell Nacreous Layer 

**Figure 2:**
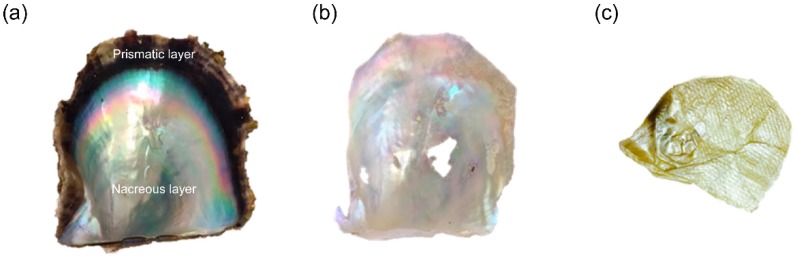
From left to right: The Japanese pearl oyster (*Pinctada fucata*) shell, the nacreous layer and the nacreous organic sheet. (**a**) Inner side of a shell. (**b**) The exposed iridescent nacreous layer following mechanical removal of the prismatic layer. (**c**) The nacreous organic sheet (NOS) was obtained following an ethylenediamine-*N*,*N*,*N*′,*N*′-tetraacetic acid (EDTA)-mediated decalcification step to remove the nacreous layer.

After the decalcification procedure, the dull-textured nacreous organic sheet (NOS) was obtained (Figure 2).

### 3.2. EEP Protein Composition

**Figure 3:**
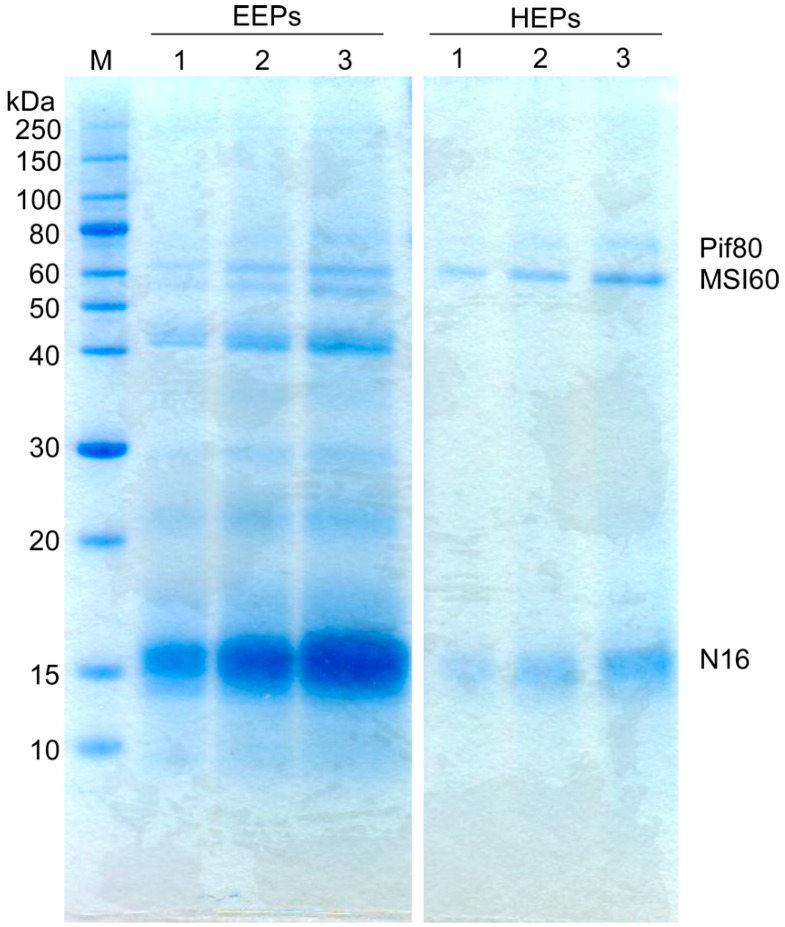
Analysis of electro-extracted (EEPs, **left**) and heat-extracted proteins (HEPs, **right**) by SDS-PAGE and Coomassie blue staining. Positions of molecular weight standards are indicated (left). M: molecular weight markers, EEPs: electro-extracted proteins, HEPs: heat-extracted proteins. Three different sample volumes were loaded on each gel. Lane 1: 5 µL, lane 2: 10 µL, lane 3: 15 µL. The positions of Pif80, MSI60 and N16 were predicted based on their relative mobilities on SDS-PAGE compared with the molecular weight markers.

Following the electroextraction step, the yield of EEPs from 30 mg of NOS was 4.38 mg. Analysis of EEPs and HEPs using SDS-PAGE and Coomassie staining revealed that the EEP samples contained considerably more protein bands compared with the HEP samples (Figure 3). Furthermore, all of the protein bands present in the HEP samples were observed in the EEP samples, strongly suggesting that the electroextraction method was considerably more effective than the heat extraction method used for NOS protein extraction.

### 3.3. Presence of Disulfide Bonds in EEPs

**Figure 4:**
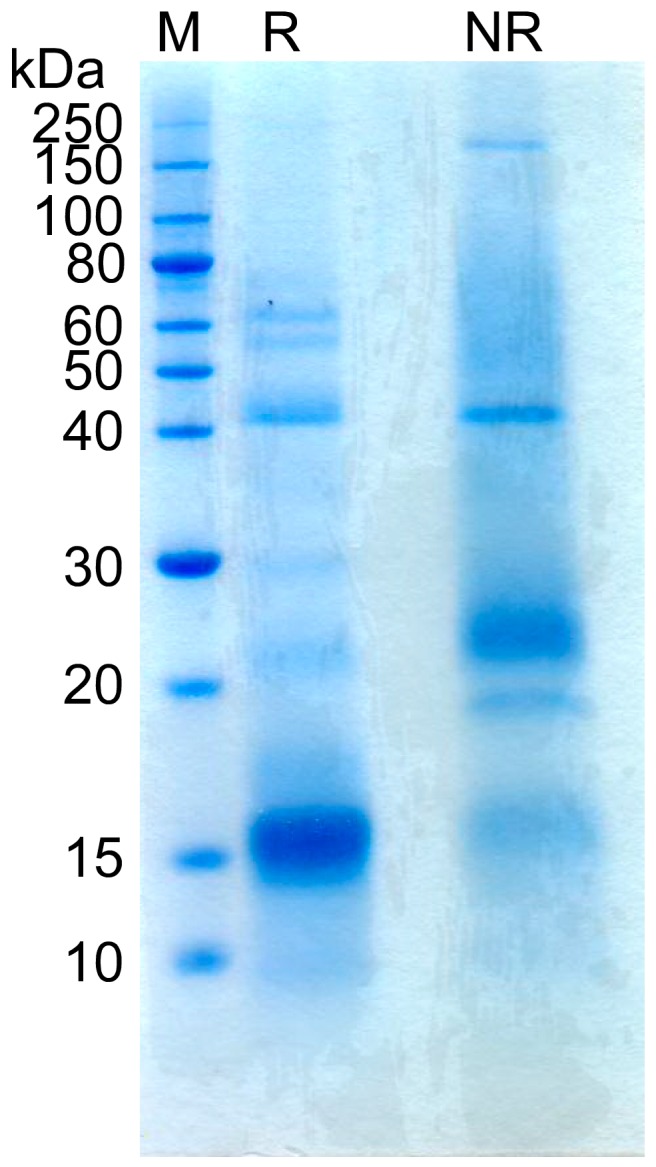
Analysis of electro-extracted proteins under reducing and non-reducing conditions using SDS-PAGE and Coomassie staining. Positions of molecular weight standards are indicated (left). M: molecular weight markers, R: reduced EEPs, NR: non-reduced EEPs.

SDS-PAGE of reduced and non-reduced EEPs showed dissimilar banding patterns (Figure 4). A major 16 kDa protein band—possibly N16—in the reduced EEP samples was not observed in the non-reduced EEPs, which suggested that the 16 kDa protein possesses intermolecular disulfide bonds. A high molecular weight protein of ~200 kDa that was only observed in the non-reduced EEP sample could be a complex of multiple subunits that contain intermolecular disulfide bonds. Furthermore, the non-reduced EEP samples contained a smaller overall number of bands compared with the reduced EEP samples, which may indicate that the NOS contains a number of protein complexes that are stabilized by intermolecular disulfide bonds.

## 4. Discussion

Here, we report a novel electroextraction method that is able to remove proteins with intact intermolecular disulfide bonds from the organic matrix of the nacreous layer. The SDS-PAGE analysis of the electro-extracted proteins suggested that additional trace components were able to be extracted from the nacreous organic sheet using our method compared with a heat treatment method performed in the presence of detergent and a reducing agent.

Most *P. fucata* secretory proteins derived from the mantle tissue contain cysteine residues [10], which suggests that they form disulfide bonds. Disulfide bonds may contribute to the rigidity and the function(s) of the nacreous layer proteins. Some soluble nacreous proteins form intermolecular disulfide bonds. P20 found in the acetic acid soluble fraction of the nacreous layer of *Pinctada maxima* can form oligomers made of six monomers linked together by disulfide bonds [11]. P60 obtained by decalcifying the nacreous layer of *P. fucata* by acetic acid is a protein complex consisting of several subunits linked by disulfide bonds [12]. P60 accelerates the differentiation of osteoblast precursor cells to earlier mineralization. It is considered that these oligomeric structures formed by intermolecular disulfide bonds are used to protect the secreted protein from degradation during the synthesis of the nacreous layer [11,12]. Thus, disulfide bonds might play important roles in the nacreous layer formation, through the formation of protein complexes. In this study, we revealed that several insoluble nacreous layer proteins contain disulfide bonds. Currently, little information is available on the relationship between insoluble nacreous layer protein disulfide bond formation and the construction of the aragonite layer. Our electroextraction method permits the isolation of insoluble nacreous layer proteins with intact intermolecular disulfide bonds, which will undoubtedly be helpful for future investigations into their functions.
